# Role of Colposcopy after Treatment for Cervical Intraepithelial Neoplasia

**DOI:** 10.3390/cancers12061683

**Published:** 2020-06-24

**Authors:** Annu Heinonen, Maija Jakobsson, Mari Kiviharju, Seppo Virtanen, Karoliina Aro, Maria Kyrgiou, Pekka Nieminen, Ilkka Kalliala

**Affiliations:** 1 Department of Obstetrics and Gynecology, University of Helsinki and Helsinki University Hospital, 00029 Helsinki, Finland; mari.kiviharju@hus.fi (M.K.); seppo.virtanen@hus.fi (S.V.); karoliina.aro@hus.fi (K.A.); pekka.nieminen@hus.fi (P.N.); ilkka.kalliala@hus.fi (I.K.); 2 Department of Obstetrics and Gynecology, Hyvinkää Hospital, HUCH and University of Helsinki, 05850 Hyvinkää, Finland; maija.jakobsson@hus.fi; 3 Department of Gut, Metabolism and Reproduction & Department of Surgery and Cancer, Institute of Reproductive and Developmental Biology, Faculty of Medicine, Imperial College London, London SW7 2AZ, UK; m.kyrgiou@imperial.ac.uk; 4 West London Gynaecological Cancer Centre, Imperial College Healthcare NHS Trust, London W2 1NY, UK

**Keywords:** colposcopy, CIN, follow-up, test of cure

## Abstract

Colposcopy is often used in follow-up after treatment for cervical intraepithelial neoplasia (CIN) despite its marked inter-observer variability and low sensitivity. Our objective was to assess the role of colposcopy in post-treatment follow-up in comparison to hrHPV (high-risk human papillomavirus) testing, cytology, and cone margin status. Altogether, 419 women treated for histological high-grade lesion (HSIL) with large loop excision of the transformation zone (LLETZ) attended colposcopy with cytology and hrHPV test at six months. Follow-up for recurrence of HSIL continued for 24 months. Colposcopy was considered positive if colposcopic impression was recorded as high grade and cytology if HSIL, ASC-H (atypical squamous cells, cannot exclude HSIL), or AGC-FN (atypical glandular cells, favor neoplasia) were present. Overall, 10 (10/419, 2.4%) recurrent HSIL cases were detected, 5 at 6 months and 5 at 12 months. Colposcopic impression was recorded at 407/419 6-month visits and was positive for 11/407 (2.7%). None of them had recurrent lesions, resulting in 0% sensitivity and 97% specificity for colposcopy. Sensitivity for the hrHPV test at 6 months was 100% and specificity 85%, for cytology 40% and 99%, and for margin status at treatment 60% and 82%, respectively. While the hrHPV test is highly sensitive in predicting recurrence after local treatment for CIN, colposcopy in an unselected population is not useful in follow-up after treatment of CIN.

## 1. Introduction

Cervical cancer develops from precursor lesions called cervical intraepithelial neoplasia (CIN), which can be treated before their progression into cancer [1]. The most common treatment modality for CIN today is a large loop excision of the transformation zone (LLETZ) [2]. Not all high-grade CIN lesions warrant treatment [3] and the number of annual CIN treatments in different countries is heavily influenced by national screening and treatment practices [4] In 2017, the incidence of histological high-grade lesions HSIL (CIN 2/3) in Finland was 74/100,000 women [5], while the incidence of annual treatments in women aged 21–30 years in the US was estimated to be over 600/100,000 women [4]. Treatment of CIN is effective in preventing subsequent invasive cervical cancer, but women treated for CIN remain at increased risk of developing new CIN compared to the general population [6]. The majority of cases develop within the first two years after treatment [7,8] and approximately 7% of treated women are diagnosed with recurrence during the 18-month follow-up period [9]. Large lesion size and increasing histopathological grade of the lesion, smoking, older age of the patient, involved cone margins, and hrHPV (high-risk human papillomavirus) persistence have been detected as risk factors that predict recurrence [10,11,12]. Furthermore, compared to the general population, the risk for cervical cancer after treatment for CIN is increased for at least 25 years [11,13,14,15].

The presence or absence of hrHPV is an accurate method to predict treatment failure or cure [9,16,17,18] also when compared to cytology and margin status [19,20,21,22]. Furthermore, affected, or positive, resection margins of the excised cone are associated with an increased risk of treatment failure [12,22], while a recent meta-analysis found the subsequent hrHPV test to be the most accurate method to predict treatment outcome [9]. 

In some countries, including Finland and Germany, colposcopy is still included in the primary follow-up in national guidelines [23,24]. However, the role and added value of colposcopy in follow-up has rarely been studied. Old studies present conflicting results [25,26,27]: Others stating that colposcopy does not significantly improve the sensitivity of cytology in early post-treatment follow-up [27] while others report 100% sensitivity for colposcopy for residual disease [25]. Additionally, colposcopy has adverse psychological effects, including anxiety, distress, and a negative effect on sexual function [28]. UK guidelines recommend hrHPV at 6 months as a test of cure (TOC) but do not present evidence on the role of colposcopy [29]. The European guidelines recommend follow-up at 6, 12, and 24 months but do not define the role of colposcopy in the follow-up [30]. The American guidelines recommend hrHPV as a TOC at six months after treatment regardless of cone margins but outlines follow-up at six months with colposcopy as acceptable, especially for those with positive margins [31]. 

HrHPV testing has been well established as the gold standard TOC after treatment of CIN, but the evidence on the value of colposcopy in the follow-up, especially when resection margins are positive, is still lacking. The aim of our study was to assess the value of colposcopy in follow-up after local excisional treatment, by comparing the diagnostic accuracy of the margin status at treatment and colposcopy, cytology, and hrHPV test at six months in predicting HSIL recurrence after local excisional treatment. Furthermore, we aimed to explore the value of colposcopy in women with positive margins. 

## 2. Results

Characteristics of the study cohort are shown in Table 1. The mean age of the patients at the time of treatment was 35.1 years (range 20.1–76.6). Smoking status at LLETZ or at 6 months was available for 393/419 (93.8%) cases, and more than one-third were smokers (143/419, 35.8%). Of the non-smokers 233/419 (64.2%) majority had never smoked (217/233, 93.1%). In 105 cases of the 419 (25.3%), LLETZ was performed at the first visit (select and treat), either due to suspicion of HSIL lesion, or due to the presence of the type 3 transformation zone (TZ3) along with a high-grade referral cytology, according to the Finnish current care guidelines [23]. Otherwise, the indication for the LLETZ procedure was persistent CIN1 (low-grade histological lesion, cervical intraepithelial lesion grade 1) in 1.7% (7/419), CIN2 (cervical intraepithelial neoplasia grade 2) for 40 % (165/419), CIN3 (cervical intraepithelial neoplasia grade 3) for 29.6% (123/419), and AIS (adenocarcinoma in situ) for 0.2% (1/419). Two patients (2/419, 0.4%) had both AIS and CIN3 in biopsies.

At the six-month visit, punch biopsies were taken of 303/419 (72.3%) of the women. At 12 months, 70 out of 419 attended a follow-up visit, 45 out of 419 at 18 months, and 298 out of 419 at 24 months, with follow-up data available. At 24 months, nearly one-third (121/419, 28.9%) were lost to follow-up. 

Overall, only 10 women (10/419, 2.4%) developed residual or recurrent histological HSIL lesions during the 24-month follow-up period. Five of them were diagnosed at six months and an additional five cases at 12 months after the LLETZ. 

The colposcopic assessment was available for 407/419 (97.1%) of the 6-month visit. Colposcopy was considered positive in 11/407 (2.7%), but none of them had HSIL histology in biopsies. Colposcopy was considered negative in 396/407 (97.3%) women at 6 months, but 9 of them 9/396 (2.3%) had HSIL in random biopsies, 5 at the 6-month visit, and 4 at the 12-month visit. For one recurrent case, the colposcopic assessment was not available (Table 2). Colposcopic suspicion of high-grade lesion performed poorly as a TOC at six months (sensitivity 0 %, specificity 97 %, PPV 0 %, and NPV 98 %) (Table 3).

A cytology result at 6 months was available for 418/419 (99.8%) women. Cytology was considered negative for 412/418 (98.6%) of patients and positive for 6/418 (1.4%) of patients. Altogether, 6/412, (1.5%) of the women with negative cytology had recurrence: One at 6 months and 5 at the 12-month follow-up visit. Of the cytology-positive women, 4/6 (57.2%) developed a recurrent disease, all of them at 6 months. (Table 2). Cytology at a cut-off of HSIL resulted in sensitivity of 40%, specificity of 99%, PPV of 67%, and NPV of 99% (Table 3). In a sensitivity analysis including all women (*n* = 491), using LSIL as the cytology threshold and LSIL (CIN1+) histology as the outcome, the cytology result was available for 490/491 (99.8%) women. Cytology was negative for 473/490 (96.5%) and 26 of them (26/473 (5.7%)) had recurrent histological LSIL. Cytology was positive for 17/490 (3.5%) women and 7/17 (41.2%) had recurrent LSIL. 

HrHPV test data were available for 407/419 (97.1%) at 6 months. Altogether, 335/407 (82.3%) women were HrHPV negative at 6 months after LLETZ and none of them developed recurrent disease during the 24-month follow-up. HrHPV was positive for 72/407 (17.7%) at 6-month visits and 9/74 (12.5%) developed recurrent disease. Four recurrent lesions were detected at 6 months and 5 at 12 months. In one recurrent case, the hrHPV test result was missing (Table 2). HrHPV testing performed best as the diagnostic test of cure, with a sensitivity of 100%, specificity of 85%, NPV of 100%, and PPV of 12% (Table 3). All women with recurrence were hrHPV positive at 6 months but colposcopic impression was negative, indicating that colposcopy performs poorly on HrHPV-positive women.

Surgical resection margins were free of HSIL for 339/419 (80.9%) of the cases. Among women with free resection margins, recurrent HSIL was detected in 4/339 (1.2%), 2 at 6 months and 2 at 12 months. Surgical margins were affected in 80/419 (19.1%) of cones and 6/80 (7.5%) had recurrence, 3 at 6 months and 3 at 12 months (Table 4). The margin status of the first cone as a predictor of recurrent lesion resulted in a sensitivity of 60%, specificity of 82%, PPV of 8%, and NPV of 99% (Table 3).

In all women, regardless of the initial histopathological grade of the treated lesion or if reoperation was done, margins were affected for 123/491 (25.0%) women. Of the affected margins, 66/123 (53.7%) were ectocervical, 37/123 (30.1%) endocervical, and 12/123 (16.3%) had both margins affected. Reoperation before the first follow-up visit was performed for 38/123 (26.8%) of those with affected resection margins. Of them, 13/38 (34.2%) had an affected ectocervical margin, 17/38 (44.7%) endocervical margin, and for 8/38 (21.1%) both margins were affected in the original cone. In reoperation cones, only 26.3% (10/38) had histological HSIL and any dysplasia was found only in 11 specimens (11/38, 28.9%). For those patients with persistent histological lesion in reoperation, endocervical margins were originally affected in 8/11 cases, ectocervical margins in 1/11, and both marginals in 2/11 cases. 

Recurrent HSIL, excluding residual HSIL still present at possible reoperation, was detected in 6/123 (4.9%) women with affected margins during the follow-up. There were 5/66 (7.6%) recurrent cases after affected ectocervical margins and 1/20 (5.0%) when both margins were affected. We detected no recurrences (0/37) among women with affected endocervical margins (Table 3). Colposcopic impression at 6 months was recorded for 121/123 of those with affected margins. Six women with affected margins, with or without immediate retreatment before the first follow-up visit, had a recurrent HSIL (6/121 5.0%). None of them were detected by colposcopic impression at six months. 

Cigarette smoking did not affect the recurrence rate, as three recurrent cases were detected (3/143 2.1%) among smokers and 6/250 (2.4%) among the non-smokers. All recurrent cases of the non-smokers were among women who had never smoked. 

## 3. Discussion

The diagnostic performance of colposcopic assessment alone at 6 months after LLETZ to predict treatment outcome, i.e., recurrence of HSIL during the follow-up was poor. HrHPV testing alone at 6 months, on the other hand, was a reliable test of cure (TOC) to predict HSIL recurrence. Affected margins were associated with recurrence but were not a good predictor of treatment failure alone. Surprisingly, colposcopy was not useful either when assessing only those women with positive resection margins. Furthermore, three quarters of repeated cones done for affected margins were free of residual disease. 

Even though colposcopy has now widely been abandoned in follow-up after LLETZ, to our knowledge, this is one of the very rare studies where the added value of colposcopy after treatment was assessed. Women treated for CIN have an increased risk of recurrence [2,11,32] and therefore follow-up protocols after treatment need to identify women at risk. HrHPV testing has been shown in several studies to be the most reliable test of cure after treatment of CIN [7,9,16,19,21,33]. Our study is in concordance with these results, with the performance of the hrHPV test at 6 months being suitable as a TOC alone to predict treatment success and to a lesser extent treatment failure. In only one recurrent dysplasia case, the hrHPV test result was missing. Here, only 2.4% (10/419) of the treated HSIL cases developed a residual or recurrent disease during the two-year follow-up period. This is a slightly lower number than in previous reports [7,32,34]. According to the Finnish Current Care Guidelines, women with negative hrHPV, cytology, and histology at 6 months are followed up with cytology and the hrHPV test at 24 months. This explains the small number of women examined at 12 and 18 months and could well explain the lower overall recurrence compared to other studies, where more intense surveillance might result in the detection of lesions that are likely to regress spontaneously [4]. 

Finland has a very good track record in preventing cervical cancer, with an efficient nationwide organized screening program since the 1963. This program includes colposcopy in a major role in diagnosis, treatment, and follow-up of cervical dysplasia. Thus, the Finnish Guidelines have been very cautious to make any major changes in the practices without good evidence. The results are based on a prospective cohort setting and recruitment from everyday clinical practice from a single referral center, thus making our results generalizable. Colposcopies were performed by certified colposcopists and all of the samples were analyzed in a single referral laboratory. 

The European guidelines recommend follow-up after treatment of CIN at 6, 12, and 24 months but do not explicitly state what should be used as the TOC or what the role of colposcopy is [30]. The American guidelines recommend hrHPV as the test of cure but state that follow-up with colposcopy is acceptable [31]. UK guidelines recommend hrHPV testing at 6 months as TOC. All guidelines refer to good results on hrHPV as a TOC but no references to the actual role or added value of colposcopy is given. In Finland, colposcopy has traditionally been used as a TOC 6 months after LLETZ, even though the inter-observer reproducibility of colposcopy is poor [35,36]. The performance of colposcopy as a test of cure after LLETZ was poor here as well, and did not reliably identify the patients that developed recurrent disease. After treatment, epithelialization or scarring of the cervix might appear, making assessment of the transformation zone unreliable and likely leading to 303 patients having random-like punch biopsies taken without apparent colposcopic impression of HSIL according to the national guidelines, which were in effect during the study. Therefore, colposcopy, as a TOC, can be both time and resource consuming and often lead to unnecessary biopsies [4]. Even though some publications have endorsed random biopsies in primary diagnostics [37,38], no evidence has been presented in follow-up. Thus, it is essential to abandon procedures and old practices that are not useful. Considering also the psychological burden and anxiety inflicted by colposcopy [28,39,40] and adverse effects of colposcopy and biopsies [41], unnecessary colposcopy could even be harmful and should not be used as the primary tool in follow-up of treated patients. The likelihood of detecting histological HSIL is much greater when performing colposcopy for HPV-positive women after treatment, whereas adverse effects are likely to dominate if colposcopy is routinely performed for all women during follow-up. 

Even though an affected margin status has been associated with treatment failure [8,12,42], some studies [43] and a recent meta-analysis [9] found that margin status is not as accurate as hrHPV to predict treatment failure. Here, the sensitivity of the margin status to predict treatment failure was 60% (95% CI 0.31–0.83), specificity 81% (95% CI 0.78–0.85), PPV 8% (95% CI 0.03–0.15), and NPV 99% (95% CI 0.97–1.00). The corresponding numbers in the meta-analysis by Arbyn et al. were a sensitivity of 55.8% (95% CI 45.8–65.5) and specificity of 84.4% (95% CI 79.5–88.4). Recurrence was highest among those with affected margins (7.5%). The corresponding number in the meta-analysis by Arbyn et al. was 17.1%. On the other hand, in the 38 women who had an immediate reoperation for affected margins, only one-third had residual HSIL still present at the repeat cone. Of the six recurrent cases with affected margins where immediate reoperation was not performed, five were hrHPV-positive at 6 months, and for one, hrHPV was not taken. All these six recurrent cases occurred in patients who had affected ectocervical margins. This is contradictory to other studies, where the probability of recurrence has been reported to be higher if the endocervical margin was affected [9,44], although in some studies the risk of recurrent disease was the same irrespective which margin was affected [42,45]. A recent register-based study reported women with involved margins to be at increased risk for recurrent disease; however, the risk was not increased, if only the ectocervical margin was affected [20]. In our study, almost half of the patients with positive endocervical margins had a repeated LLETZ versus 21 % of the cases who had an affected ectocervical margin, but our total numbers here are low and conclusions should be prudent. This might explain the lower recurrence with affected endocervical margins observed here. Still, most women who had immediate reoperation due to affected resection margins did not present residual HSIL at the reoperation. Furthermore, all women with a positive margin status and recurrent disease were hrHPV positive at 6 months. Furthermore, the histological assessment of surgical margins might be hampered due to the thermal effect of the LLEZT, resulting in interpreting a complete excision as incomplete [46]. On the other hand, even with clear margins, residual disease might still be present within endocervical crypts or as skip lesions [15]. This warrants a much more conservative approach towards reoperations for women with affected margins. Follow-up with the hrHPV test and restriction of colposcopy for HPV-positive women only might be an acceptable approach here as well. Repeated LLETZ procedures should perhaps be restricted to cases where there is histological confirmation of residual disease or persistent hrHPV positivity after the treatment along with affected margins, and these conclusions are supported by previous literature [9,20]. 

Our study has some limitations. Patients attended follow-up visits well, but we did lack some follow-up data, reflecting the true clinical practice. The hrHPV status at 6 months was known for 116/121 of the women lost to follow-up at 24 months and 71.6% (83/116) of them were negative and 28.4% (33/116) positive, whereas overall only 17.7% of all women were hrHPV positive at six months. This might well lead to underestimation of the recurrence rate as women who were hrHPV positive at six months were both at increased risk of recurrence as well as to loss to follow-up and perhaps explain the lower recurrence observed here compared to some previous publications [7,9]. The follow-up lasted for two years, before returning to the 5-year interval screening program. This duration of intensified follow-up is generally considered to be sufficient, but still, a longer follow-up would be needed to rule out late recurrences. The relatively high number of reoperations conducted on 38/123 women with positive margins may also have influenced the total recurrence rate. However, in only 10/38 of these women, CIN2+ was still present at reoperation and even when including these cases, the rate of residual and recurrent cases would have been 4.4% (20/457). Additionally, in total, our numbers of different positive margin statuses of ecto vs. endo are low and hence caution should be used to draw conclusions.

## 4. Materials and Methods 

**Figure 1:**
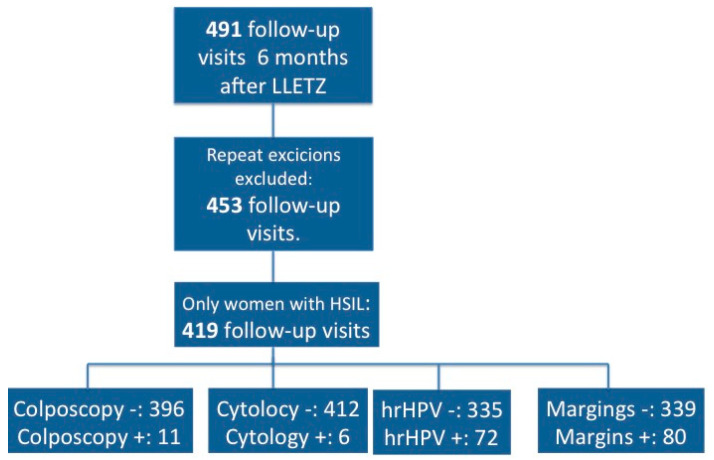
Flowchart.

This study was a part of a prospective cohort study (HELICOPTER study: ISRCTN10933736) conducted at Helsinki University Hospital’s Outpatient Colposcopy Unit, Finland, between January 2014 and May 2016. The unit serves as the sole reference center in the Helsinki-Uusimaa region with over 1.6 million residents. In this study, we included all women treated with LLETZ with at least six months of follow-up after treatment (*n* = 491). The main study population comprised of women with no repeated excisions and HSIL as the worst histology (*n* = 419) (Figure 1).

Women were followed up according to the Finnish Current Care guidelines. All treated women were invited to the first colposcopy follow-up visit at six months, where cytological sample, hrHPV test, and colposcopy guided biopsies of suspected HSIL were taken. In case no HSIL was suspected, random biopsies were taken at the discretion of the individual colposcopist. If the cytology, histology, and hrHPV were negative at six months, the next control visit comprised an hrHPV test and cytology at 24 months. If hrHPV was positive at 6 months, patients were recalled for colposcopy at 12 months and again at 18 months if hrHPV positivity persisted [23]. Of the women who attended the 18-month visit, 22/45 were hrHPV negative at 6 months and most of these later visits were due to additional vulvar lesions present at the time of CIN treatment or previous follow-up visits.

The hrHPV samples were analyzed initially using Hybrid Capture II (Digene Corporation, MD, USA) and since April 2015 the Messenger RNA-based Aptima® assay (Hologic, Inc, MA, USA). Performance of both tests has been proven to be similar in the detection of high-grade lesions [47]. The test was considered positive if any of the HPV genotypes were present. Cytology samples were traditional Papanicolaou smears and all excised cones were reviewed by histopathologists specialized in gynecological histopathology. 

Recurrence was defined as histologically confirmed HSIL (CIN2 or CIN3) or more severe lesion at the 6-, 12-, or 24-month follow-up visits. Colposcopy was considered to be positive if colposcopic impression, assessed by the colposcopist performing the procedure, was of high grade (HSIL), regardless of whether histopathological samples (punch biopsies) were taken or not. Cytology was considered to be positive if HSIL (high-grade squamous intraepithelial lesion), ASC-H (atypical squamous cells, cannot rule out HSIL), or AGC-FN (atypical glandular cells, favor neoplasia) was detected. The margins status of the excised cone was considered either free (negative) or affected (positive), depending on whether the HSIL lesion extended to endocervical, ectocervical, or both margins. If the margin status was reported as unclear, we considered the margins to be affected.

The performance of the hrHPV test, cytology, and colposcopy and cone margin status as tests of cure were assessed in terms of their sensitivity, specificity, negative predictive value (NPV), and positive predictive value (PPV) to detect recurrence within the follow-up. Differences in recurrence proportions between different groups were assessed using Fisher’s exact test. 

We repeated analyses including women with repeated excisions and all women regardless of histopathological finding (*n* = 491), with additional sensitivity analysis using LSIL as a threshold for cytology and histology. The performance of the margin status as a predictor of recurrence was also analyzed separately for all women irrespective of histology and further stratified according to whether reoperation was done or not. If an immediate reoperation was done for affected margins and HSIL persisted in the second cone, persistent HSIL was not considered as a recurrent lesion. All results were also additionally stratified according to smoking status.

All statistical analyses were done with IBM SPSS statistics (version 24). The study was approved by Helsinki University’s Ethical Committee (130/13/03/03/2013, 24.4.2013) and written informed consent was acquired from all participants.

## 5. Conclusions

In conclusion, colposcopy was not useful primarily in the follow-up of patients after treatment for CIN—overall or when assessing patients with affected surgical margins only. The HrHPV test alone at 6 months is an accurate test of cure and colposcopy should be reserved for patients who are hrHPV positive after treatment. Routine repeated LLETZ procedures due to affected cone margins should be avoided and those patients primarily managed with active surveillance instead.

## Figures and Tables

**Table 1:** Demographics.

Main (*n* = 419)				All (*n* = 491)			
Mean age (SD 8.4)	35.1 (20.1–76.6)			Mean age (SD 9.3)	36.3 (20.1–76.6)		
	*n* (%)				*n* (%)		
Smoking				Smoking			
Yes	143 (34.1)			Yes	168 (34.2)		
No	250 (59.7)			No	293 (59.7)		
NA	26 (6.2)			NA	30 (6.1)		
Total	419 (100.0)			Total	491 (100.0)		
Indication for LLETZ		Worst histology	*n* (%)	Indication for LLETZ		Worst histology	*n* (%)
Persistent LSIL/CIN1	7 (1.7)	CIN2	5 (71.4)	Persistent LSIL/CIN1	8 (1.6)	CIN1	1 (12.5)
		CIN3	2 (28.6)			CIN2	5 (62.5)
						CIN3	2 (25)
HSIL/CIN2	165 (40.0)	CIN2	121 (73.3)	HSIL/CIN2	165 (34.0)	CIN1	0 (0.0)
		CIN3	44 (26.7)			CIN2	118 (71.5)
						CIN3	47 (28.5)
HSIL /CIN3	123 (29.6)	CIN2	2 (1.6)	HSIL/CIN3	134 (27.6)	CIN1	0 (0.0)
		CIN3	121 (98.4)			CIN2	1 (0.7)
						CIN3	133 (99.3)
AIS	1 (0.2)	AIS	1 (100)	AIS	3 (0.6)	CIN1	0 (0.0)
						CIN2	0 (0.0)
						CIN3	1 (33.3)
						AIS	3 (100)
CIN3 and AIS	2 (0.5)	CIN3	2 (100)	CIN3 and AIS	2 (0.4)	CIN1	0 (0.0)
		AIS	1 (50)			CIN2	0 (0.0)
						CIN3	2 (100)
						AIS	1 (50)
Select andtreat *	105 (25.3)	CIN2	37 (35.2)	Select and treat *	154 (31.7)	CIN1	7 (4.5)
		CIN3	68 (64.8)			CIN2	41 (26.6)
		AIS	1 (1.0)			CIN3	89 (57.8)
						AIS	6 (39.0)
						Other **	15 (9.7)
Other *****	12 (2.9)	CIN2	7 (58.2)	Other ***	20 (4.1)	CIN1	2 (10)
		CIN3	5 (41.7)			CIN2	7 (35)
		AIS	2 (16.7)			CIN3	4 (20)
						AIS	3 (15)
						Other ****	5(25)
NA	5(1.2)			NA	5/1.2)		
Total	419 (100.0)			Total	491 (100.0)		

SD, standard deviation, LLETZ large loop excision of the transformation zone, LSIL/CIN1, low-grade squamous intraepithelial lesion, grade 1, AIS adenocarcinoma in situ, NA not available. * select and treat (cytology ASC-H (atypical squamous cells, cannot exclude HSIL), HSIL (high-grade squamous intraepithelial lesion) or AGC-FN (atypical glandular cells, favor neoplasia) and colposcopic impression of HSIL or TZ3 (transformation zone type 3)) ** Normal histology 8, LSIL 6, adenocarcinoma 1 *** 11 Discrepancies, 2 referral cytology + histology, 1 postcoital bleeding, 6 atypia in columnar cells **** 5 LSIL ***** 2 atypia in columnar cells, 7 discrepancies, 1 post-coital bleeding, 2 referral cytology + histology.

**Table 2:** Recurrence of HSIL for women with HSIL as worst histology, repeat LLEZT excluded.

*n*/N (%) *	6 Months *n*/N (%)	12 Months *n*/N (%)	18 Months *n*/N (%)	24 Months *n*/N (%)	All Cumulative *n*/N (%)
Overall HSIL (*n* = 419)	5/419(1.2)	5/70 (6.6)	0/45 (0.0)	0/298 (0.0)	10/419 (2.4)
Colposcopy at 6 months ** (*n* = 407)	(*n* = 407)	(*n* = 66)	(*n* = 42)	(*n* = 288)	(*n* = 407)
+11/407 (2.7)	0/11 (0.0)	0/3 (0.0)	0/0 (0.0)	0/7 (0.0)	0/12 (0.0)
−396/407 (97.3)	5/396 (1.3)	4/63 (6.3)	0/42 (0.0)	0/267 (0.0)	9/396 (2.3)
Cytology at 6 months *** (*n* = 418)	(*n* = 418)	(*n* = 70)	(*n* = 43)	(*n* = 284)	(*n* = 418)
+6/418 (1.4)	4/6 (66.7)	0/4 (0.0)	0/0 (0.0)	0/2 (0.0)	4/6 (57.2)
−412/418 (98.6)	1/412 (0.2)	5/66(7.6)	0/43 (0.0)	0/282 (0.0)	6/412 (1.5)
HrHPV at 6 months (*n* = 407)	(*n* = 407)	(*n* = 69)	(*n* = 39)	(*n* = 277)	(*n* = 407)
+72/407 (17.7%)	4/72 (5.6)	5/58 (8.6)	0/17 (0.0)	0/31(0.0)	9/72 (12.5)
−335/407 (82.3)	0/335 (0.0)	0/11 (0)	0/22 (0.0)	0/246 (0.0)	0/355 (0)

* *n* = cases of histological HSIL, N = cases assessed ** Colposcopy was considered positive if colposcopic assessment was HSIL *** Cytology was considered positive is ASC-H (atypical squamous cells, cannot exclude HSIL), HSIL (high-grade cervical intraepithelial lesion) or AGC-FN (atypical grandular cells, favor neoplasia) was present. LLETZ large loop excision of the transformation zone

**Table 3:** Comparison of different tests of cure at 6 months and margin status at LLEZT to predict recurrence of HSIL lesions at 24-month follow-up. Recurrence of HSIL overall at 24 months was 2.4%.

Test of Cure	Sensitivity (95% CI)	Specificity (95% CI)	PPV (95% CI)	NPV (95% CI)
Colposcopy	0.00 (0.00–0.30)	0.97 (0.95–0.98)	0.00 (0.00–0.24)	0.98 (0.96–0.99)
Cytology	0.40 (0.17–0.69)	0.99 (0.98–1.00)	0.67 (0.30–0.94)	0.99 (0.97–0.99)
HrHPV	1.00 (0.70–1.00)	0.85 (0.81–0.88)	0.12 (0.07–0.22)	1.00 (0.99–1.00)
Margins	0.60 (0.31–0.83)	0.82 (0.78–0.85)	0.08 (0.03–0.15)	0.99 (0.97–1.00)

PPV = positive predictive value, NPV = negative predictive value CI confidence interval, HrHPV high-risk human papillomavirus.

**Table 4:** Recurrence of HSIL lesions during the 24-month follow-up after LLETZ: according to the resection margin status of the first LLETZ and separately for all women regardless for histopathology and according to whether reoperation for affected margins was performed.

Margins Affected	Margin Status	6 Months *n*/N (%) *	12 Months *n*/N (%)	18 Months *n*/N (%)	24 Months *n*/N (%)	All Cumulative *n*/N (%)
Any Margin						
No re-LLETZ, only HSIL (*n* = 419)	Free (*n* = 339)	2/339 (0.6)	2/51 (3.9)	0/28 (0.0)	0/236 (0.0)	4/339 (1.2)
	Affected (*n* = 80)	3/80 (3.8)	3/19 (15.8)	0/15 (0.0)	0/47 (0.0)	6/80 (7.5)
All LLETZ (*n* = 491)	Free (*n* = 368)	2/368 (0.5)	2/56 (3.6)	0/36 (0.0)	0/249 (0.0)	4/368 (1.1)
	Affected (*n* = 123)	3/123 (2.4)	3/26 (11.5)	0/23 (0.0)	0/74 (0.0)	6/123 (4.9)
No re-LLETZ	Affected (*n* = 85)	2/85 (2.4)	3/20 (15)	0/16 (0.0)	0/469(0.0)	5/85 (5.8)
Re-LLETZ	Affected (*n* = 38)	1/38 (2.6)	0/6 (0.0)	0/7 (0.0)	0/25 (0.0)	1/38 (2.6)
Ectocervical margins (66/123)						
All LLETZ	Affected	2/66 (3.0)	3/12 (25)	0/15 (0.0)	0/40 (0.0)	5/66 (7.6)
No re-LLETZ	Affected	2/53 (1.9)	3/10 (30)	0/11 (0.0)	0/29 (0.0)	4/52 (7.7)
Re-LLETZ	Affected	0/13 (0.0)	0/2 (0.0)	0/4 (0.0)	0/11 (0.0)	1/14 (7.1.)
Endocervical margins (37/123)						
All LLETZ	Affected	0/37 (0.0)	0/9 (0.0)	0/5 (0.0)	0/24 (0.0)	0/37 (0.0)
No re-LLETZ	Affected	0/20 (0.0)	0/7 (0.0)	0/3 (0.0)	0/14(0.0)	0/20 (0.0)
Re-LLETZ	Affected	0/17 (0.0)	0/3 (0.0)	0/2 (0.0)	0/10 (0.0)	0/17 (0.0)
Both Margins (20/123)						
All LLETZ	Affected	1/20 (5)	0/3 (0.0)	0/3 (0.0)	0/10 (0.0)	1/20(5.0)
No re-LLETZ	Affected	1/12 (9.1)	0/2 (0.0)	0/2 (0.0)	0/6 (0.0)	1/11 (9.1)
Re-LLETZ	Affected	0/8 (0.0)	0/1 (0.0)	0/1 (0.0)	0/4 (0.0)	0/9 (0.0)

* *n* = cases of histological HSIL (high-grade cervical intraepithelial lesion), N = cases assessed, LLETZ large loop excision of the transformation zone.
